# Cognitive improvement effects of electro-acupuncture for the treatment of MCI compared with Western medications: a systematic review and Meta-analysis

**DOI:** 10.1186/s12906-018-2407-2

**Published:** 2019-01-08

**Authors:** Hwan Kim, Hong Kyoung Kim, Si Yeon Kim, Young Il Kim, Ho Ryong Yoo, In Chul Jung

**Affiliations:** 1 Department of Oriental Neuropsychiatry, College of Korean Medicine, College of Oriental Medicine, Daejeon University, Yongun-dong, Dong-gu, Daejeon, South Korea; 2 Clinical Trial Center, Dunsan Korean Medicine Hospital of Daejeon University, Daejeon, South Korea; 3 Department of Acupuncture and Moxibustion, College of Oriental Medicine, DaeJeon University, Daejeon, South Korea; 4 Department of Circulatory Internal Medicine, College of Oriental Medicine, DaeJeon University, Daejeon, South Korea

**Keywords:** Mild cognitive impairment (MCI), Electroacupuncture (EA), Randomised controlled trials (RCT), Systematical reviews (SR), Mini mental, State examination (MMSE), Montreal cognitive assessment (MoCA)

## Abstract

**Background:**

Almost half of mild cognitive impairment (MCI) patients progress to dementia, which is associated with decreased quality of life and obstacles to independent living. Relevant management is expected to prevent MCI patients from progressing to dementia. In recent years, electroacupuncture (EA) has been used to treat various kinds of neurological disorders including MCI. This study evaluates the use of EA for MCI patients to increase cognitive function through a comparison with Western medications.

**Methods:**

Randomized controlled trials (RCT) or systematical reviews (SR) of EA versus Western medications for MCI were searched using the following 10 databases: Pubmed, Cochrane Library, CINAHL, EMBASE, China National Knowledge Infrastructure (CNKI), National Digital Science Library (NDSL), Journal of Oriental Neuropsychiatry (JON), Korean Medical Database (KMBASE), KoreaMed, and OASIS, from October 2007 to August 2017, without language restriction. A methodological quality assessment of RCTs or SRs that met inclusion criteria was conducted using Cochrane Risk of bias (RoB) tool and a meta-analysis by RevMan (Review Manager) 5.3.5 version of Cochrane collaboration.

**Results:**

Five RCTs with 257 patients met inclusion criteria and those were randomly divided into two groups: the EA group (*n* = 103) and Western medications group (*n* = 154). The methodological quality of the included studies showed high risk or/and unclear of risk of bias. The meta-analysis of five studies reported that the EA group was better than the Western medications group, improving the Mini Mental State Examination (MMSE) score by 0.65 [95% CI 0.28~1.01] higher mean difference, Montreal Cognitive Assessment (MoCA) score by 0.66 [95% CI 0.00~1.32] higher mean difference. Adverse effects were not reported in the selected studies.

**Conclusion:**

Electroacupuncture was an effective treatment for MCI patients by improving cognitive function. However, the included studies presented a low methodological quality and no adverse effects were reported. Thus, further comprehensive studies with a design in depth are needed to derive significant results.

## Background

Mild Cognitive Impairment (MCI) refers to cognitively impaired at memory section but not clinically demented yet [1]. Patients with MCI generally go through this transition before progressing to dementia. Almost half of MCI patients tend to progress to fulfill a diagnostic criteria of dementia within five years [2, 3]. According to statistical results from the National Health Insurance Service (NHIS) of the Republic of South Korea, the number of people who visited hospitals as MCI patients increased from 24,000 to 105,000 between 2010 and 2014. This indicates that the number of patients increased 43.9% every year and became 4.2 times greater within four years. This increasing pattern was similar to that observed for cases of dementia [4]. The ratio of MCI patients to Alzheimer’s Disease(AD) patients was expected to increase by more than one-fourth [4]. Patients with MCI have a high possibility to progress to dementia but are not yet at the stage of irreversible dementia. Relevant management is expected to increase the possibility of MCI patients progressing to AD [5]. As the numbers of dementia patients are increasing rapidly and cognitive impairment is the most common symptom of MCI, management should be primarily focused on this aspect [6, 7].

Acupuncture has been used as one of the main treatments in the clinical field from thousands of years ago. Furthermore, elderly people are comfortable with this treatment. EA treatment refers to inserting more than two needles into the skin and applying weak electricity through the needle. This gives the patients an acupuncture effect and electricity stimulation simultaneously. Recently, many studies have been performed to evaluate EA and the results generally showed efficacy and safety [8] for neurological diseases such as MCI [9], schizophrenia [10], vascular dementia [11, 12], stroke [13], depressive symptoms [14], Parkinson’s disease [15], and Alzheimer’s disease [16]. However, a systematical review of studies on MCI has not been performed to date.

The aim of the present study is to perform a systematical review to evaluate the efficacy and safety of EA in terms of improving cognitive function in comparison with Western medications. If the results of this study demonstrate that EA improves cognitive function, they would greatly influence not only Oriental medical intervention therapy but also the Korean clinical field and even national policy.

## Methods

### Literature search

The PICO-SD (Participants, Intervention, Comparison, Outcome, Study Design) form was used to compose key questions. MCI patients (participants) were treated with EA (Intervention) or Western medications (Comparison) to evaluate the effect of EA on cognitive function (Outcome).

Researchers searched the literature on MCI patients to compare the effect of EA to Western medications from the following major and additional databases from May 2017 to August 2017: The major databases were Pubmed, Cochrane Library, CINAHL, EMBASE, and the Chinese database China National Knowledge Infrastructure (CNKI). Additional databases from Korea were the National Digital Science Library (NDSL), Journal of Oriental NeuroPsychiatry (JON), Korean Medical Database (KMBASE), KoreaMed, and OASIS. Publication status was from inception to August 20, 2017 without language restriction.

Combinations of search words from major databases consisted of MCI (Mild Cognitive Impairment), cognitive disorder, amnesia, memory impairment, CDR 0.5, and mild neurocognitive disorder’ for participants and electroacupuncture, and electric acupuncture’ for intervention (Appendix 1, 2, 3 and 4), Chinese databases were searched with mild cognitive impairmentʼ and 'electroacupuncture (Appendix 5). Additional (Korean) databases were searched with mild cognitive impairment, cognitive impairment, cognitive disorder, and electroacupuncture (Appendix 6, 7). Searched studies from each database were chosen according to inclusion and exclusion criteria by reading the title and abstract as the first selection and reading the full-text for the final selection.

### Selection criteria of studies

Randomized controlled trials (RCT) and Systematical Review (SR) of study designs were selected. Included studies were comparing EA group to Western medications group in terms of improving cognitive function as the outcome for mild cognitive impairment (MCI) patients.

### Selection criteria of participants

No restriction on age, sex, nation, race, period of treatment or contraction of disease, economical state, inpatient or outpatient of MCI. To include natural cases of primary MCI patients, specific diseases or situations such as vascular mild cognitive impairment (vMCI), diabetic mild cognitive impairment, mild cognitive impairment by Parkinson’s disease, and mild cognitive impairment by surgery were excluded. The patients who fulfilled diagnostic criteria of dementia were excluded.

The most prevalently used standard diagnosis criteria were 《Diagnostic&Statistical Manual of Mental Disorder 4th (DSM—IV)》 from West and 《Standards of Chinese medical syndrome differentiation for deficient syndrome》 from China [17, 18]. Standard diagnosis criteria were not restricted.

### Selection criteria of interventions

Combined intervention with Western medications, herbs, moxibustion, and intensified training of cognitive ability were excluded. Also, the usual acupuncture treatment skills found in Korean acupuncture textbooks and steadily used in the clinical field were included and specific acupuncture treatment skills (Table 1) that were not usually used in the clinical field were excluded.

**Fig. 1:**
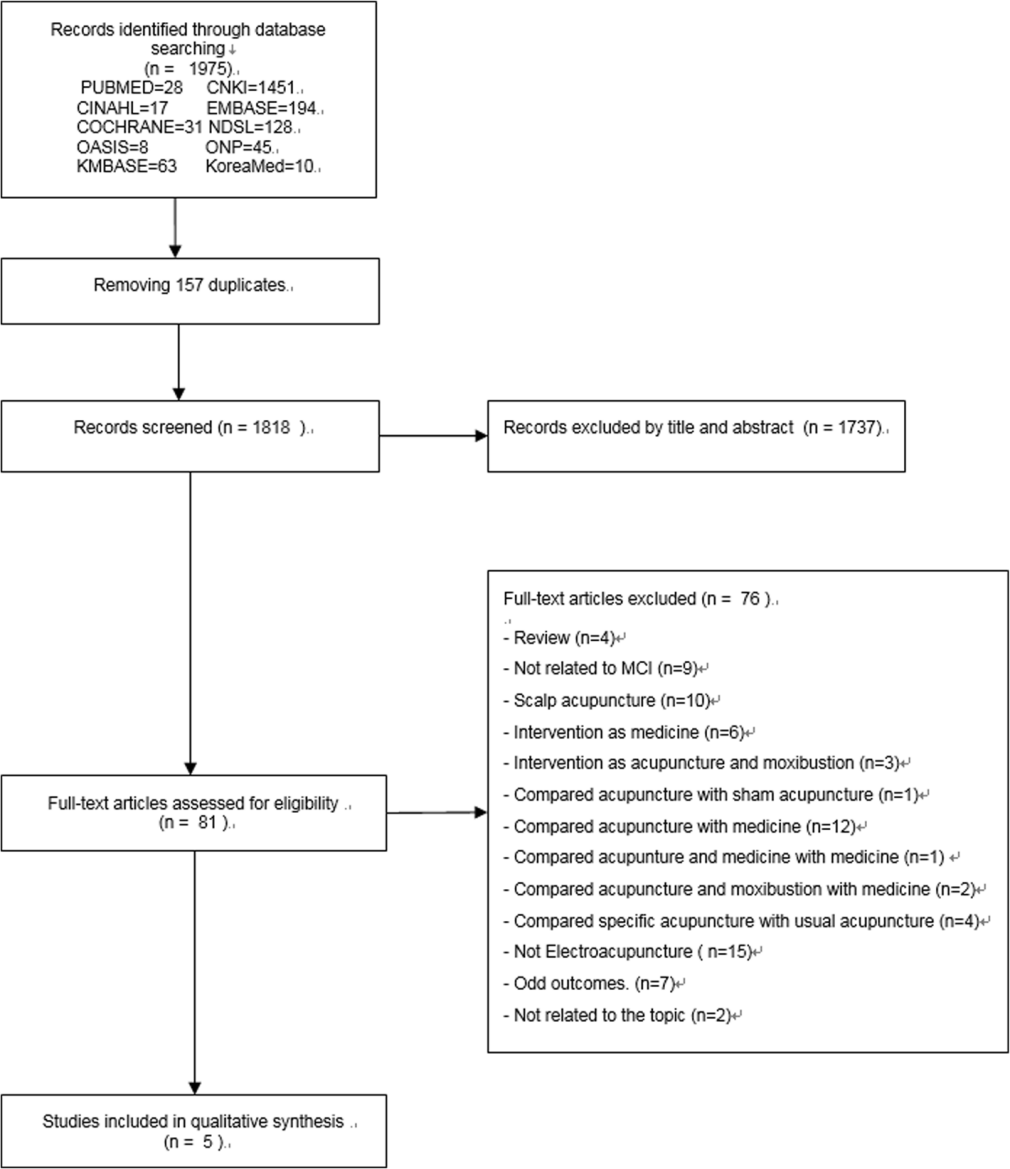
Flow-chart of studies included in the meta-analysis on effect of electroacupuncture to MCI patients

**Table 1:** Examples of excluded acupuncture treatment skills

- Jin three needle therapy	
- Scalp acupuncture	
- Three temporal points	
- Treatment alternately daily at front and rear Sishenchong (EX-HN1) + right Fengchi (GB20)/left and right Sishenchong (EX-HN1) + Shenting (DU24), Baihui (GV20)	
- Retaining needle time for 30 min alternately daily at left and right Sishenchong (EX-HN1)/Shenting (DU24), Baihui (GV20) + front and rear Sishenchong (EX-HN1)	
- Twirling reinforcing and reducing method or mild reinforcing attenuating needle pricking therapy	
- Twirling reinforcing and reducing method or back and forth moving manipulations	
- Electroacupuncture after de-qi sensation by twirling method at sachonghyeol (SCH; Four command points), Yintang (EX-HN3), Fengchi (GB20)	
- Electroacupuncture after twirling and back and forth moving manipulations at Wailaogong (EX-UE8), Fenglong (ST40) San yin jiao (SP6) Taixi(KI3)	
- Applying acupuncture in specific sequence	
- Implement twirling reinforcing and reducing method differently	
- Putting needle 30° at Indang (Ex-HN3), 45° at Taiyang (EX-HN5), directing to end of nose at Fengfu (DU16), Baihui (gv20). Back and forth moving manipulations at FengFu (DU16), Fengchi (GB20) Hegu (LI4) Taichong (LR3)/twirling method at Indang (Ex-HN3), Baihui (GV20)	
- Electroacupuncture + mild reinforcing attenuating needle pricking therapy

**Table 2:** Summary table of five papers

First Author (year)	Study Design	Patients	Gender (male/female), age (years), duration of disease (years or months)		Intervention	Comparison	Duration of Treatment	Outcomes	Results	Adverse effect
			Intervention (3 arms are with two intervention)	Comparison						
Zhao (2012)	RCT	(1) Meets MCI diagnostic criteria of “Expert Consensus on Prevention and Treatment of Cognitive Impairment in China”. (2) Main symptoms of memory impairment. (3) Other cognitive impairments are relatively low. (4) Does not affect the ability of daily living. (5) Does not meet the diagnosis criteria for dementia. (6) Does not cause other brain functional decline. (7) GDS (Global Deterioration Scale) 2–3. (8) CDR (Clinical Dementia Rating) 0.5, 1.5SD (Standard Deviation) or lower than the average result of memory test compared to the same age and education level onset over three months. (9) MMSE 24–27. (10) 55–85 years. (11) Scheduled education level or higher, ≥1 year	Intervention 1 -(−/−) 73.35±4.60 - Intervention 2 -(−/−) 72. 68 ± 8. 26	-(−/−) 72.96±4.95	electroacupuncture alone (EX-HN1,GV20,GV24,GB20)(28) electroacupuncture and syndrome differentiation (EX-HN1,GV20,GV24,GB20+ KI03, ST40 SP10, LR03, ST36)(28)	anti-dementia drugs (Nimodipine) (28)	2 months	① MMSE	① MMSE 1) electroacupuncture alone: 25.50 ± 0.81 to 27.93 ± 1.41. 2) electroacupuncture and syndrome differentiation.: 25.46 ± 0.82 to 28.13 ± 1.22. 3) anti-dementia drug (Nimodipine): 25.28 ± 0.85 to 27.43 ± 1.38	not reported
Sun(2010)	RCT	(1) If symptoms of memory decline exceeds 3 months. (2) Objective evidence of memory decline. ‘Neuropsychological index score < 1.5 standard deviation of the mean value of normal elderly’. (3) If we cannot diagnose dementia based on DSM, we cannot diagnose it because of senile dementia based on NINCDS-ADRDA.	Intervention 1 20(10/10) 68±9 26.65 ± 21.17 (months). Intervention 2 20(10/10) 67 ± 9 29.90 ± 26.30 (months)	20(10/10) 68±929.05 ± 23.63 (months)	electroacupuncture alone (GV20,GV24,GB13(Electroacupuncture) + BL18,BL23,PC06,LI04,KI06,LR03) (20 electroacupuncture (GV20,GV24,GB13(Electroacupuncture),BL18,BL23,PC06,LI04,KI06,LR03) + anti-dementia drugs (donepezil)) (20)	anti-dementia drugs (donepezil)) (20)	30 days	① MMSE ② MoCA	① MMSE 1) electroacupuncture alone: 21.95 ± 2.33 to 24.95 ± 1.99 2) electroacupuncture + anti-dementia drugs (donepezil)): 22.40 ± 2.60 to 28.80 ± 0.62 3) anti-dementia drugs (donepezil)): 22.45 ± 2.44 to 25.10 ± 1.68 ② MoCA 1) electroacupuncture alone: 12.70 ± 2.89 to 18.60 ± 2.28 2) electroacupuncture + anti-dementia drugs (donepezil)): 12.55 ± 3.02 to 23.20 ± 1.54 3) anti-dementia drugs (donepezil)): 12.90 ± 2.73 to 18.20 ± 3.38	Not reported
Liu(2009)	RCT	(1) DSM-IV standard (2) < Chinese medicine syndrome differentiation reference standard》 diagnosis standard (17)	8(−/−) 73±8 2.50±0.93 (years)	9(−/−) 77±6 2.33±0.66 (years)	electroacupuncture alone (EX-HN1, GB20, BL23, HT07, GB39, KI03) (8)	anti-dementia drugs (Donepezil) (9)	1 months	① MMSE ② CMS	① MMSE (After treatment). 1) electroacupuncture alone: 28.63 ± 1.69. 2) anti-dementia drugs (Donepezil): 27.11 ± 1.45. ② CMS (After treatment) 1) electroacupuncture alone: 84.50 ± 13.46 2) anti-dementia drugs (Donepezil): 70.28 ± 19.06	not reported
Liu(2010)	RCT	(1) MCI diagnosed on DSM-IV criteria. (2) MMSE score according to educational background (No education > 18, Elementary School graduation > 21, middle school graduation > 24). (3) GDS 2~3. (4) CDR 0.5. (5) ADL < 26. (6) Memory loss is insufficient to diagnose dementia. (7) "Criteria for the diagnosis of 《Chinese medicine syndrome differentiation reference standard》. (8) 60 ~ 85 years old	17(7/10) 66.00±6.84 2.500±0.9258 (years)	19(9/10) 69.32±6.86 2.320±0.6614 (years)	electroacupuncture alone (GV20, BL23, GB39, GB20, KI03) (17)	anti-dementia drugs (Donepezil) (19)	1 months	① MMSE	① MMSE. 1) electroacupuncture alone: 24.52 ± 2.87 to 28.25 ± 1.91. 2) anti-dementia drugs (Donepezil): 24.03 ± 3.16 to 27.23 ± 2.92	not reported
Xu(2017)	RCT	(1) Meet MCI diagnosis criteria (2) 65–80 years (3)Revised 2008 NINCDS-ADRDA diagnosis criteria (4) Good basic physical strength, chest X and blood, Brain MRI examination clear	30(16/14). 62.12±8.01 8.55±3.10 (months)	30(16/14) 61.20±7.63 9.60±2.72 (months)	electroacupuncture alone (GV20, GV24, EX-HN1, GB20) (30)	anti-dementia drugs (Nimodipine) (30)	2 months	①MMSE ②MoCA ③ CDT ④ P300 ⑤ ERP (hs-CRP,IL-6) ⑥ MRI	① MMSE. 1) electroacupuncture alone: 24.49 ± 0.62 to 27.72 ± 1.49. 2) anti-dementia drug (Nimodipine): 24.49 ± 0.62 to 26.95 ± 1.34 ② MoCA 1) electroacupuncture alone: 24.53 ± 0.60 to 27.70 ± 1.50 2) anti-dementia drug (Nimodipine): 24.05 ± 0.11 to 27.00 ± 1.30 ③ CDT. 1) electroacupuncture alone: 3.10 ± 0.25 to 3.45 ± 0.33. 2) anti-dementia drug (Nimodipine): 3.12 ± 0.30 to 3.30 ± 0.51 ④ P300. 1) electroacupuncture alone: 449.80 ± 14.28 to 397.84 ± 15.43 (Incubation period): 3.49 ± 0.30 to 4.69 ± 0.22 (covering). 2) anti-dementia drug (Nimodipine): 448.66 ± 14.14 to 408.64 ± 15.27 (Incubation period): 3.56 ± 0.28 to 4.28 ± 0.27 (covering). ⑤ Reduction of ERP (hs-CRP, IL-6). ⑥ Local consistency of functional magnetic resonance imaging (MRI) of left temporal lobe, left hippocampus, occipital lobe, tongue, and anterior wedge.	not reported

**Fig. 2:**
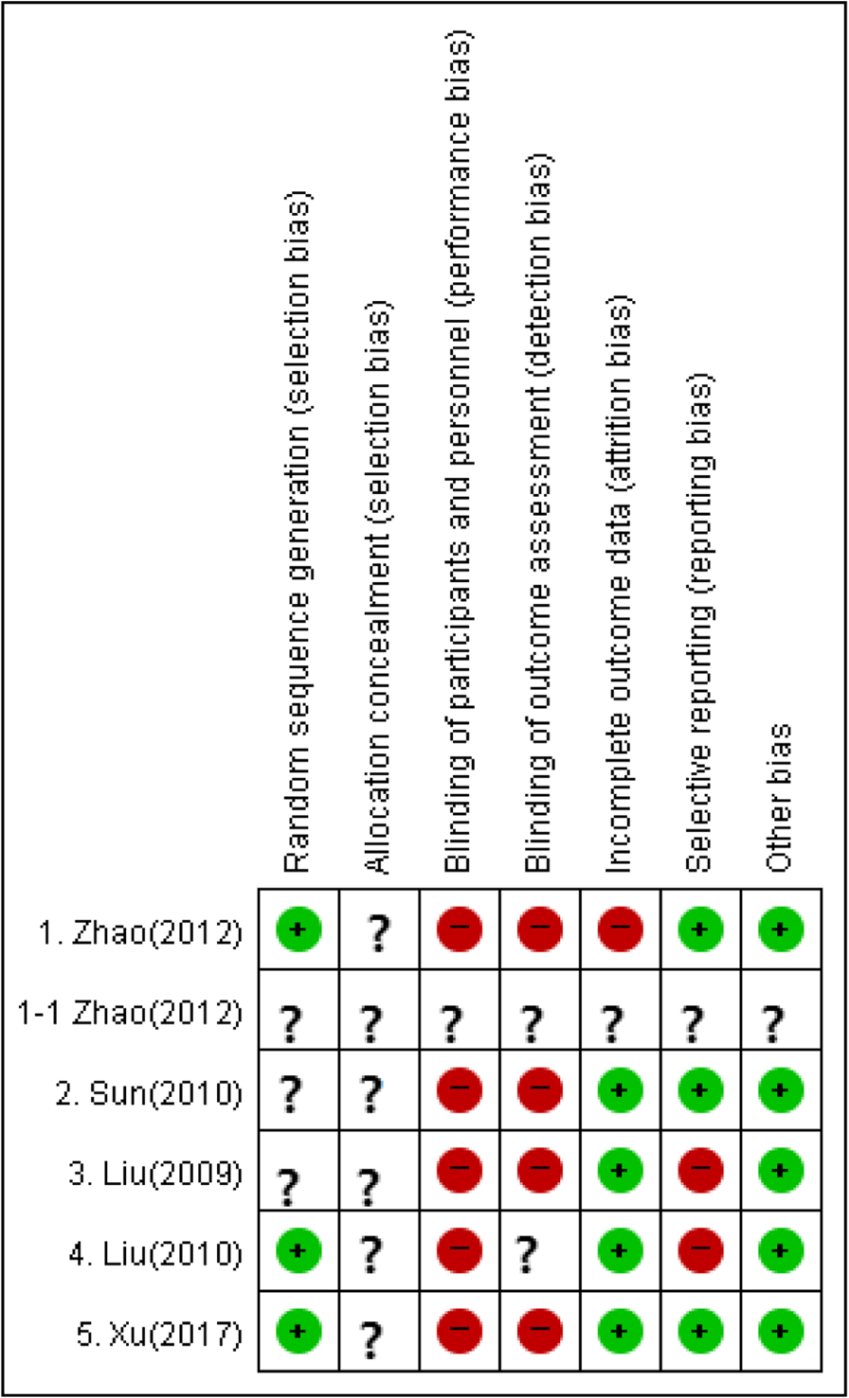
Risk of bias of studies included in the meta-analysis on effect of electroacupuncture to MCI patients

**Fig. 3:**
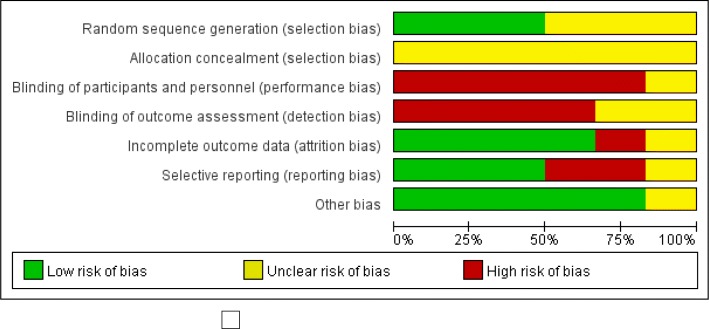
Risk of bias of studies included in the meta-analysis on effect of electroacupuncture to MCI patients

**Fig. 4:**
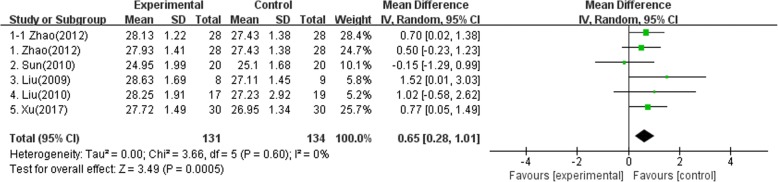
Meta-analysis of MMSE of studies included (1–1: electroacupuncture and syndrome differentiation group vs. anti-dementia drugs group, 1–2: electroacupuncture alone group vs anti-dementia drugs group)

**Fig. 5:**
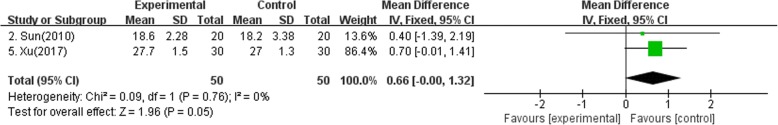
Meta-analysis of MoCA of studies included

### Selection criteria of comparatives

Comparison was standard treatment of Western medication. Other Oriental medical treatments (e.g., sham acupuncture, usual acupuncture, herbs, moxibustion) were excluded., if it had Western medication comparison among the case of having two comparisons, was included in the data synthesis.

### Selection criteria of outcomes

MMSE and MoCA were used as outcome to evaluate the effect on improving cognitive function. In other words, the remaining outcomes except those two were excluded from the data synthesis.

### Data extraction

The overall processes of data collection and extraction were performed independently by two researchers (HK, HKK) according to approved inclusion criteria. Conflicting opinions were discussed to reach agreement. ICJ coordinated conflicting opinions between researchers.

The final five studies met inclusion criteria and were arranged by code according to the features of the literature. Categories of coding graphs were writer, publishing date, study design, selection criteria of participants, number of participants, intervention period, comparison treatment, tool of outcomes, result of outcomes, acupoints used in intervention treatment, and reported adverse effects.

### Quality assessment

A quality assessment of the final selected five studies was performed independently by two researchers (HK, HKK) using the risk of bias (ROB) tool of Cochrane. All of the assessing evidence was admitted only when the exact context was expressed in the literature. Conflicting opinions were discussed to reach agreement. ICJ coordinated the conflicting opinions between researchers.

### Data synthesis and analysis

A meta-analysis was carried out using RevMan (Review Manager) version 5.3.5, and the results were summarized by a Forest plot. Since the papers included in this study are continuous data, mean and standard deviation (SD) values were used.

The meta-analysis focused on the evaluation tools commonly used to evaluate the effectiveness of cognitive function improvement (ex. MoCA, MMSE). For the comparison to the two control groups, the analysis was conducted according to researcher agreement and a meta-analysis manual for the 3-arm RCT of the Cochrane alliance.

In the meta-analysis, the fixed-effect model and random-model are used. In the case of less than three data, the fixed model was used. In the case of more than four data, one of them was determined considering the statistical heterogeneity.

In order to examine the statistical heterogeneity, the evaluation method was homogeneity verification. If I-Squared is 0% or the *P*-value is larger than 0.1, the statistical heterogeneity is low and the fixed model can be used. If I-Squared is not 0%, there is statistical heterogeneity and a random model can be used. However, since the magnitude of the effects of the literature included in this study may be different and there is clinical heterogeneity, a random model was used instead of the fixed model, even if the homogeneity was verified.

1. MMSE: It was proved that the statistical heterogeneity was low because of I-Squared = 0% *p* = 0.60, But a random model was used in consideration of clinical heterogeneity.
2. MoCA: A fixed model was used because there are less than three data.

## Results

### Literature search

A total of 1975 papers were searched in databases using search terms and search strategies (Fig. 1). In the study, 1818 subjects, excluding 157 duplicates, were selected as subjects for the study. The titles and abstracts were reviewed, and the results were as follows: "Animal experiments, physiology / pathology / pharmacology studies, review papers, observational studies, cohort studies, survey, pilot study, protocol, preliminary study, expert opinion, patient irrelevant (patients with other diseases, healthy subjects), intervention method irrelevant (bilateral drug treatment, unrelated to oriental medicine); out-of-paper formats (comment, note, reply, news article, editorial, summaries of conferences, etc.) were excluded, and a total of 1737 were excluded. After reviewing the original texts of 81 selected papers, the final five papers in line with key questions [19–23] were selected.

### Description of included studies

A total of five papers were randomized controlled clinical trials (RCT) and published in China in 2009, 2012, and 2017, and two in 2010, respectively.

In Zhao (2012) [19], a total of 84 patients were treated with electroacupuncture alone, electroacupuncture and syndrome differentiation treatment, and anti-dementia drugs (Nimodipine). In the electroacupuncture and syndrome differentiation group, additional selection of points with the electroacupuncture point was made on the basis of syndrome differentiation according to the scale for the differentiation of syndromes. After two months, the MMSE score was 27.93 ± 1.41 for the electroacupuncture alone group and 27.43 ± 1.38 for the anti-dementia group. The difference between the two groups was statistically significant (*p* < 0.05). On the other hand, the MMSE of the electroacupuncture and syndrome differentiation group was 0.70 (95% CI: 0.02, 1.38) higher than that of the anti-dementia group.

In Sun (2010) [20], a total of 60 patients were treated with electroacupuncture alone, combination therapy (electroacupuncture, anti-dementia drugs (donepezil)), and anti-dementia drugs (donepezil). After 30 days, the MMSE of the electroacupuncture alone group was 0.15 (95% CI: -1.29, 0.99) lower than that of the anti-dementia group. The MoCA of the electroacupuncture alone group was 0.40 (95% CI: -1.39, 2.19) higher than that of the anti-dementia group. The difference between the two groups was not statistically significant (*p* > 0.05). On the other hand, the MMSE of the combination therapy group was 3.70 (95% CI: 2.92, 4.48) higher than that of the anti-dementia drug group. The MoCA of the combination therapy group was 5.00 (95% CI: 3.37, 6.63) higher than that of the anti-dementia group.

In Liu (2009) [21], a total of 17 patients were treated with electroacupuncture alone and anti-dementia drugs (donepezil). After one month, the MMSE of the electroacupuncture alone group was 1.52 (95% CI: 0.01, 3.03) higher than that of the anti-dementia drug group. The CMS (Clinical Memory Scale) score was 84.50 ± 13.4 for the electroacupuncture alone group and 70.28 ± 19.06 for the anti-dementia drug group. The difference between the two groups was statistically significant (*p* < 0.05).

In Liu (2010) [22], a total of 36 patients were treated with electroacupuncture alone and anti-dementia drugs (donepezil). After one month, the MMSE of the electroacupuncture alone group was 1.02 (95% CI: -0.58, 2.62) higher than that of the anti-dementia drug group. The difference between the two groups was not statistically significant (*p* > 0.05).

In Xu (2017) [23], a total of 60 patients (65–80 years) were treated with electroacupuncture alone and anti-dementia drugs (Nimodipine). After two months, the MMSE of the electroacupuncture alone group was 0.77 (95% CI: 0.05, 1.49) higher than that of the anti-dementia drug group. The MoCA of the electroacupuncture alone group was 0.70 (95% CI: -0.01-1.41) higher than that of the anti-dementia drug group. The difference between the two groups was not statistically significant (*p* > 0.05). On the other hand, the CDT (clock drawing task) score was 3.30 ± 0.51 for the electroacupuncture alone group and 3.45 ± 0.33 for the anti-dementia drug group. The difference between the two groups was statistically significant (*p* < 0.05).

### Patient characteristics

The five studies were quantitatively synthesized. The total number of participants was 257, 103 in the intervention group and 154 in the control group.

### Intervention

The most commonly used acu-points employed in the intervention method (electroacupuncture) were GV20, GB20 (four papers), followed by EX-HN1, GV24, KI03, BL23 (three papers), GB39, LR03 (two papers), and ST40, SP10, ST36, GB13, BL18, PC06, LI04, KI06 and HT07 (one paper). The duration of the treatment was one month (three papers) and two months (two papers).

### Outcome measures

A commonly reported assessment tool is MMSE (five papers), MoCA (two papers). In one paper, CMS was reported and in another paper, CDT, E1A-associated 300 kDa protein (P300), event-related potential (ERP), high sensitivity C-reactive protein (hs-CRP), and interleukin-6 (IL-6) were reported. To confirm the improvement of cognitive function, a meta-analysis was conducted on MMSE and MoCA, which were reported by many RCTs. In all five papers, adverse reactions were not reported.

### Control conditions and treatment of 3-arms study

Among the five papers, in two studies (Zhao (2012), Sun (2010)), two control groups were established, and the total number of control group was seven (Donepezil 3, Nimodipine 2, Electroacupuncture and syndrome differentiation treatment 1, Electroacupuncture and donepezil 1).

Zhao (2012) was a comparative study of the treatment group of electroacupuncture alone, electroacupuncture and syndrome differentiation group, and anti-dementia drugs (Nimodipine). According to the agreement from researchers and the meta-analysis manual for 3-arm RCT of the Cochrane Alliance, this study was divided into two parts (electroacupuncture alone versus anti-dementia drugs (Nimodipine), electroacupuncture and syndrome differentiation treatment versus anti-dementia drugs (Nimodipine)) and the effects were compared with the electroacupuncture and the anti-dementia drug, and then a meta-analysis was conducted in the same way. Finally, two sets of data were obtained from this paper.

Sun (2010) was a comparative study of the treatment group of electroacupuncture alone, electroacupuncture and anti-dementia drugs (Donepezil), and anti-dementia drugs (Donepezil). In the study, the best results were obtained with a combination of electroacupuncture and anti-dementia drugs, but this study focused on a comparison between the effects of the electroacupuncture and anti-dementia drugs in this study.

In other words, a total of six data were used for the meta-analysis in order to compare the effectiveness of the treatment of electroacupuncture and anti-dementia drugs (Table 2).

### Risk of bias

The RoB tool was used to evaluate the risk of bias in five selected papers (Fig. 2). The quality of the included studies was generally low or unclear, but was high for blinding.

Three of five papers used a random number table, and thus the risk of bias was evaluated as “Low” (Fig. 3) in the random sequence generation section.

In the allocation concealment section, there was no clear description of all five sections, and thus the risk of bias was evaluated as “Unclear.”

In the blinding of participants and personnel section, because it is a comparison of the treatment of electroacupuncture and anti-dementia drugs, it is impossible to blind the participants and researchers. All five papers were evaluated as having a high risk of bias.

In the blinding of the outcome assessment section, in Zhao (2012), “The evaluator did not understand the correlation between the treatment method and the data, but only participated in the statistical part.” However, since the blinding of the patient had not already been done, risk of bias was evaluated as “Unclear”, and the remaining four papers were evaluated as “high”.

In the incomplete outcome data section, Zhao (2012) reported two missing patients in the electroacupuncture alone group, three missing patients in the electroacupuncture and syndrome differentiation group, and two missing patients in the anti-dementia drugs group, respectively. However, Zhao (2012) did not report the reason for the dropout, and the bias risk was rated as “high”. There were no cases of Sun (2010) and Xu (2017). Liu (2009) was randomly assigned to 19 selected patients after elimination of 7 out of 26 patients. In Liu (2010), 14 patients dropped out of the treatment but as the reasons for aborting or discontinuing therapy were reported, all of them were evaluated as “Low”.

In the selective reporting section, the risk of bias was evaluated as “High” because the results of the Activities of Daily Living (ADL) mentioned earlier were not described in the cases of Liu (2009) and Liu (2010), and in the remaining three cases, the risk of bias was evaluated as “Low”.

### Meta analysis

A meta-analysis (or quantitative synthesis) of the results of the MMSE was used as the evaluation variable in all five papers. According to the agreement from researchers and the meta-analysis manual for the 3-arm RCT of the Cochrane Alliance, we analyzed the six kinds of comparing categories including the results of the electroacupuncture alone group vs. the anti-dementia drugs group and electroacupuncture and syndrome differentiation group vs. the anti-dementia drugs group in 3-arm RCT Zhao (2012) study.

In addition, MoCA was reported in two papers and used in the meta-analysis. In CMS and CDT, only one paper was reported, and P300 and ERP were excluded from the meta-analysis because it was not an indicator for cognitive function improvement.

Final selected randomized controlled trials associated with MCI showed that the MMSE score was 0.65 (95% CI 0.28 to 1.01) higher and the MoCA score was 0.66 [95% CI -0.00 to 1.32 points] higher in the electroacupuncture alone group than in the anti-dementia drugs group.

1. MMSE (Fig. 4).
2. MoCA (Fig. 5).

### Safety

Adverse events were not reported in any of the papers.

## Discussion

MCI is relatively milder than dementia. However, almost half of the MCI patients have a tendency to progress to fulfill the diagnostic criteria of dementia within five years [2, 3]. Thus prevention at the stage of MCI is important. There is no medications approved by the U.S Food and Drug Administrations(FDA) for MCI patients [24]. A number of researches report that donepezil [25] and nimodipine [26] have efficacy on MCI patients. However, many people feel insufficiency by these medications and they tend to use alternative and complementry therapies including EA to treat MCI [27]. Recently, EA treatment has been widely used in clinical fields [28, 29]. Therefore, we have to evaluate which intervention between EA treatment and Western medications have more efficacy and safety. However, a systematical review to evaluate the improvement of cognitive function based on a comparison with Western medications has not been performed to date.

Thus, our research team searched many kinds of databases including China National Knowledge Infrastructure (CNKI) because Chinese databases include many studies about the effect of electroacupuncture on MCI patients. Five studies were included from searching and meta-analyzed with MMSE (*n* = 265) and MoCA (*n* = 100) as outcomes. From the results, the EA group had a higher score for the improvement effect on MMSE and MoCA.

MMSE is a major sensitive marker for not only MCI diagnosis but also dementia diagnosis. [30, 31] Also, high sensitivity and specificity were shown to distinguish MCI and dementia [32, 33]. MoCA also shows high sensitivity and specificity for diagnosis of MCI [34]. One study expressed MoCA as a more sensitive marker than MMSE for diagnosis of MCI [35]. Exact mechanisms of EA were not proven yet but EA is reported to be efficient in several ways according to animal studies on the molecular and cellular mechanisms of EA. From this evidence, we could infer the mechanisms of how it affects the patients. EA treatment reduced inflammation, oxidative stress, and apoptosis after an experimental study with septic brain injury rats and cognitive impairment rats induced by limb ischemia-reperfusion [36, 37]. EA treatment reduced inflammatory cytokines in the hippocampus, prolonged the necrosis period in pyramidal cells, and decreased activation of Acetylcholine Esterase(AChE) at vascular dementia rats [38, 39]. EA treatment upregulated the autophagy pathway, downregulated the Notch signaling pathway, and increased adenosine monophosphate-activated kinase (AMPK), which is deeply correlated to cognitive function as a master energy sensor in Alzheimer disease rats [40–42]. EA treatment had a preventative effect on cognitive impairment after brain irradiation, which was a decreasing factor of quality of life [43]. Likewise, EA was effective in treating various kinds of diseases and a common function was to control the mechanisms that cause problems with cognitive function. Controlling inflammation, stress, enzyme, delivery pathway, and antioxidant and specific enzymes that directly activate the hippocampus could be inferred as factors underlying mechanisms by which EA treatment affects cognitive impairment and prevents a decline of cognitive function simultaneously.

To evaluate the safety of EA, 5 RCTs were analyzed but no adverse effect was reported. However, to analyze other studies about the safety of EA, a few adverse effects were reported and that adverse effects even were unclear to have relevancy with EA [44]. Thus, EA appears to be a safe treatment.

Our review has important limitations that should be carefully considered. First, the methodological quality of the studies has weaknesses. According to the analysis results of RoB, all of the studies showed high risk on blinding of participants and blinding of personnel. Blinding of outcome assessment showed high risk except for one study. The score of MMSE and MoCA might be affected by the unblinding assessor. So the effect of EA on MCI might be overestimated.

Also, random sequence generation was done in three studies and showed low risk but the remaining two studies and all studies of allocation concealment were unclear. Thus, a placebo effect may have influenced the results and researchers may have violated to take neutral attitude during research. Second, the number of selected studies was low and consequently we could not access publication bias. Third, the selected papers are disproportionally focused on Chinese studies. As Chinese relatively favor EA, the results of the studies may have bias. Fourth, all of the selected studies treated patients with the same intervention of EA. However, the specific acupoints differed between the studies. The main acupoints are similar overall in the studies but various acupoints were used in individual studies. Also, as Zhao (2012) treated patients by categorizing the patients by syndrome differentiation, it becomes harder to ensure consistency of these studies.

Based on correcting these restrictions and problems, high quality studies have to be performed in the future. Researches should perform patient allocation, random sequence generation and allocation concealment with conscientious manner to keep fairness of the results. To obtain exact results, unifying acupoints of intervention therapy according to a consistent protocol is necessary. As safety is one of the most important factors to evaluate treatment, broad information is required to secure not only effective, but also trusted treatment. Overall, a large number of rigorously designed studies should be performed in the future.

## Conclusions

1. Five RCT studies on MCI patients (participants) being treated with EA (Intervention) or Western medications (comparison) to evaluate the effect of EA on cognitive function (outcome) are included.
2. The EA group had a higher score for MMSE and MoCA than the Western medications group, thus demonstrating the effect of EA on improving cognitive function.

## Appendix 1

**Table 3:** Search strategy used in Pubmed

No	Search items
1	MCI[Title/Abstract]
2	(nMCI or aMCI or mMCI).[Title/Abstract]
3	(“N-MCI” or “A-MCI” or “M-MCI”).[Title/Abstract]
4	amnestic[Title/Abstract]
5	(cognit* impair*)[Title/Abstract]
6	(cognit* declin*)[Title/Abstract]
7	(cognit* deficit*)[Title/Abstract]
8	(cognit* disturb*)[Title/Abstract]
9	“cognitive disorders”[Title/Abstract]
10	(cognit* defect*)[Title/Abstract]
11	“Cognitive defect”[Title/Abstract]
12	“Cognition Disorders”[Mesh]
13	“Amnesia”[Mesh]
14	“memory impairment”[Title/Abstract]
15	CDR AND 0.5[Title/Abstract]
16	“clinical dementia rating” AND 0.5[Title/Abstract]
17	dement*[Title/Abstract]
18	“mild neurocognitive disorder”[Title/Abstract]
19	“Subjective memory complaints”[Title/Abstract] OR SMC[Title/Abstract]
20	OR 1–19
21	“electro acupunctur*”[Title/Abstract]
22	electroacupunctur*[Title/Abstract]
23	electro-acupunctur*[Title/Abstract]
24	electric acupuncture[Title/Abstract]
25	OR 21–24
26	#20 AND #25

## Appendix 2

**Table 4:** Search strategy used in Cochrane Library

No	Search items
1	MeSH descriptor: [Cognition Disorders] explode all trees
2	MeSH descriptor: [Amnesia] explode all trees
3	MCI:ti,ab,kw (Word variations have been searched)
4	‘cognit* impair*’:ti,ab,kw (Word variations have been searched)
5	‘cognit* declin*’:ti,ab,kw (Word variations have been searched)
6	‘cognit* deficit*’:ti,ab,kw (Word variations have been searched)
7	‘cognit* disturb*’:ti,ab,kw (Word variations have been searched)
8	‘cognit* defect*’:ti,ab,kw (Word variations have been searched)
9	‘memory impairment’:ti,ab,kw (Word variations have been searched)
10	“CDR 0.5” or “clinical dementia rating scale 0.5”:ti,ab,kw (Word variations have been searched)
11	dement*:ti,ab,kw (Word variations have been searched)
12	“mild neurocognit* disorder*”:ti,ab,kw (Word variations have been searched)
13	SMC or ‘subjective memory complaints’:ti,ab,kw (Word variations have been searched)
14	#1 or #2 or #3 or #4 or #5 or #6 or #7 or #8 or #9 or #10 or #11 or #12 or #13
15	electroacupunct*:ti,ab,kw or electro-acupunct*:ti,ab,kw (Word variations have been searched)
16	#14 and #15

## Appendix 3

**Table 5:** Search strategy used in CINAHL

No	Search items
1	TI MCI OR AB MCI
2	TI “cognit* impair*” OR AB “cognit* impair*”
3	TI “cognit* declin*” OR AB “cognit* declin*”
4	TI “cognit* deficit*” OR AB “cognit* deficit*”
5	TI “cognit* disturb*” OR AB “cognit* disturb*”
6	TI “cognit* defect*” OR AB “cognit* defect*”
7	MH “Cognition Disorders”
8	TI ““memory impairment”” OR AB ““memory impairment””
9	TI ((“CDR 0.5” or “clinical dementia rating scale 0.5”)) OR AB ((“CDR 0.5” or “clinical dementia rating scale 0.5”))
10	TI dement* OR AB dement*
11	TI “mild neurocognit* disorder*” OR AB “mild neurocognit* disorder*”
12	TI (SMC or ‘subjective memory complaints’) OR AB (SMC or ‘subjective memory complaints’)
13	OR 1–12
14	TI electroacupuncture OR AB electroacupuncture
15	TI electro-acupuncture OR AB electro-acupuncture
16	OR 14–15
17	#13 AND #16

## Appendix 4

**Table 6:** Search strategy used in EMBASE

No	Search items
1	MCI:ab,ti
2	(nMCI or aMCI or mMCI).ti,ab.
3	(“N-MCI” or “A-MCI” or “M-MCI”).ti,ab.
4	amnestic:ab,ti
5	‘cognit* impair*’:ab,ti
6	‘cognit* declin*’:ab,ti
7	‘cognit* deficit*’:ab,ti
8	‘cognit* disturb*’:ab,ti
9	‘cognit* defect*’:ab,ti
10	‘cognitive defect’/exp
11	‘memory disorder’/exp
12	‘cognitive disorders’/exp
13	‘anterograde amnesia’/exp
14	‘retrograde amnesia’/exp
15	‘transient global amnesia’/exp
16	‘memory impairment’:ab,ti
17	(CDR NEAR/3 0.5):ab,ti OR (‘clinical dementia rating’ NEAR/3 0.5):ab,ti
18	dement*:ab,ti
19	‘mild neurocognit* disorder*’:ab,ti
20	‘subjective memory complaints’:ab,ti OR SMC:ab,ti
21	OR 1–20
22	‘electroacupuncture’/exp
23	‘electroacupuncture needle’/exp
24	electroacupoint*:ab,ti
25	electroacupunctur*:ab,ti
26	OR 22–25
27	#21 AND #26

## Appendix 5

**Table 7:** Search strategy used in CNKI

No	Search items
1	AB = 认知功能障碍
2	AB = 神经认知紊乱
3	AB = 神经认知损害
4	AB = 轻度认知障碍
5	AB = 神经认知障碍
6	OR 1–5
7	TX = 电针
8	TX=electro-acupuncture
9	TX=electroacupuncture
10	TX=electric acupuncture
11	OR 7–10
12	#6 AND #11

## Appendix 6

**Table 8:** Search strategy used in NDSL, OASIS, KMBASE (No.2–7), JON (No.1–7)

No	Search items
1	TI: dementia
2	TI: mild cognitive impairment
3	TI: cognitive impairment
4	TI: cognitive disorder
5	OR 1–4
6	TI: electroacupuncture
7	#5 and #6

## Appendix 7

**Table 9:** Search strategy used in Koreamed

No	Search items
1	MCI [TI]
2	“cognitive disorders” [TI]
3	“Cognition disorder” [TI]
4	(cognit* [TI] AND impair* [TI])
5	OR 1–4
6	“electroacupuncture” [TI]
7	“Clinical Trial” [PT]
8	“Controlled Clinical Trial” [PT]
9	“Meta-Analysis” [PT]
10	“Randomized Controlled Trial” [PT]
11	OR 7–10
12	“Humans” [MH]
13	#5 AND #6 AND #12

## Abbreviations

AChE: Acetylcholineesterase
AD: Alzheimer’s disease
ADL: Activities of Daily Living
AMPK: Adenosine monophosphate-activated kinase
CDT: Clock drawing task
CMS: Clinical memory scale
DSM-IV: Diagnostic & Statistical Manual of Mental Disorder 4th
EA: electroacupuncture
ERP: event-related potential
hs-CRP: high sensitivity C-reactive protein
IL-6: interleukin-6
MCI: Mild Cognitive Impairment
MMSE: Mini Mental State Examination
MoCA: Montreal Cognitive Assessment
P300: E1A-associated 300 kDa protein
RCT: Randomised controlled trials
ROB: Risk of bias
SR: Systematical Reviews
vMCI: Vascular mild cognitive impairment
WHO: World Health Organization
